# Retraction: Decreased microRNA-182-5p helps alendronate promote osteoblast proliferation and differentiation in osteoporosis via the Rap1/MAPK pathway

**DOI:** 10.1042/BSR-2018-0696_RET

**Published:** 2024-08-05

**Keywords:** ADCY6, Alendronate, MicroRNA-182, Osteoblasts, Osteoporosis, Rap1/MAPK signaling pathway

This article is being retracted from *Bioscience Reports* at the request of the Editor-in-Chief and the Editorial Board following receipt of a notification from a reader, alerting the Editorial Board to multiple concerns surrounding the scientific validity of this paper. Specifically:

- Figure 1A OP and ALN panels which seem to appear as Figure 6E and Figure 6F in a previously published paper by Han et al, 2018 (DOI: 10.1002/jcb.26478) [retracted]
- Concerning features of the Western blots, including the shape of the bands and minimal background details
- The veracity of the flow cytometry data in Figure 9

Additionally, the Editorial Office have concerns regarding alterations of images used in Figure 1A, Figure 2A, and Figure 4 that were introduced during the manuscript revision process. The authors have been contacted with regards to the retraction and have not responded to the Journal's queries or the concerns raised. Given the extent of the issues raised, the Editorial Board stand by the decision to retract the article.

